# Mice with Catalytically Inactive Cathepsin A Display Neurobehavioral Alterations

**DOI:** 10.1155/2017/4261873

**Published:** 2017-01-04

**Authors:** O. Y. Calhan, V. Seyrantepe

**Affiliations:** Department of Molecular Biology and Genetics, Izmir Institute of Technology, Gulbahce Mahallesi, Urla, Izmir, Turkey

## Abstract

The lysosomal carboxypeptidase A, Cathepsin A (CathA), is a serine protease with two distinct functions. CathA protects *β*-galactosidase and sialidase Neu1 against proteolytic degradation by forming a multienzyme complex and activates sialidase Neu1. CathA deficiency causes the lysosomal storage disease, galactosialidosis. These patients present with a broad range of clinical phenotypes, including growth retardation, and neurological deterioration along with the accumulation of the vasoactive peptide, endothelin-1, in the brain. Previous in vitro studies have shown that CathA has specific activity against vasoactive peptides and neuropeptides, including endothelin-1 and oxytocin. A mutant mouse with catalytically inactive CathA enzyme (*CathA*^*S*190*A*^) shows increased levels of endothelin-1. In the present study, we elucidated the involvement of CathA in learning and long-term memory in 3-, 6-, and 12-month-old mice. Hippocampal endothelin-1 and oxytocin accumulated in *CathA*^*S*190*A*^ mice, which showed learning impairments as well as long-term and spatial memory deficits compared with wild-type littermates, suggesting that CathA plays a significant role in learning and in memory consolidation through its regulatory role in vasoactive peptide processing.

## 1. Introduction

Cathepsin A (CathA) is a ubiquitously expressed component of a lysosomal multienzyme complex (LMC) with independent protective and catalytic functions [1, 2]. CathA is a member of the serine protease family of enzymes, with carboxypeptidase activity at pH 5.5 and deamidase and esterase activity at pH 7.0 [3]. CathA protects sialidase 1 (also known as Neu1) and *β*-galactosidase (*β*-Gal) glycosidases against proteolytic degradation by the composition of the LMC and activates Neu1. The complex is unable to form in the absence of CathA [4]. In vitro studies have shown that CathA has catalytic activity against several vasoactive peptides, including endothelin-1, angiotensin-1, and bradykinin, and some neuropeptides, such as oxytocin and substance P [5]. Genetic mutations in the CathA gene are characterized by the occurrence of the lysosomal storage disorder galactosialidosis, which involves a secondary deficiency of Neu1 and *β*-Gal [6]. It has also been demonstrated that CathA is involved in the inactivation of the lysosome-associated membrane protein type 2a (lamp2a) in chaperone-mediated autophagy [7].

The effects of immunization to CathA on learning in a rat model inspired us to investigate whether catalytically inactive CathA has similar effects on behavior and memory [8]. Although the *CathA*^−/−^ knockout mouse model presents phenotypes similar to human patients with galactosialidosis, severe symptoms arising due to the secondary deficiency of Neu1 and *β*-Gal prevent the use of this mouse model for behavioral analysis [9]. Therefore, in this study, we used a previously generated *CathA*^*S*190*A*^ knock-in mouse model with a mutation in the catalytic site. Although catalytic enzyme activity is abolished in *CathA*^*S*190*A*^ mice, they are still able to form the LMC and activate Neu1 [10].

In this study, we analyzed the performance of *CathA*^*S*190*A*^ mice in learning- and memory-based tasks at three different ages. We observed decline in cognitive functions as well as in learning and memory in *CathA*^*S*190*A*^ mice. After revealing the involvement of CathA in these behavioral changes, we investigated the levels of endothelin-1 and oxytocin in the *CathA*^*S*190*A*^ mouse brain. We demonstrated here for the first time that the hippocampal region has highly elevated levels of the vasoactive peptides: endothelin-1, angiotensin-I, and bradykinin. Our findings clearly indicate that the neurobehavioral alterations in these mice are likely the result of impaired processing of the vasoactive peptides.

## 2. Methods

### 2.1. Animals

The generation of mice containing a Ser190Ala point mutation in the CathA active site (*CathA*^*S*190*A*^ strain) was previously described [10]. The original *CathA*^*S*190*A*^ mice were provided by AV Pshezhetsky (University of Montreal, Canada). *CathA*^*S*190*A*^ mice were mated with C57BL/6 strain for at least five generations to stabilize the genetic background. Littermates were used as controls. Mice were housed in an accredited vivarium at constant temperature and humidity under 12 h light : dark cycle in accordance with the animal center's guidelines. Mice had free access to food and water. Approval for the animal care and experiments was granted by the Animal Care and Use Committee of the Izmir Institute of Technology.

### 2.2. Genotyping

All mice were genotyped for the S190A mutation using genomic DNA extracted from the tail. The PCR for the mutation was performed as previously described [10] with allele specific primers using 5′-GGTGGCGGAGAACAATTATG-3′ and 5′-AACAGAAGTGGCACCCTGA C-3′.

### 2.3. Behavioral Analysis

#### 2.3.1. Passive Avoidance Test

Both male wild-type (*n* = 7) and *CathA*^*S*190*A*^ (*n* = 8) mice at 3, 6, and 12 months old of age were subjected to the passive avoidance test. The test was conducted as previously described [11, 12]. The apparatus consisted of a two-compartment box, a lit compartment connected to a dark compartment by a guillotine door. When the animal stepped into the dark compartment with all four paws, the door was closed and a 0.2 mA foot shock was delivered for 1 s. The latency times for entering the dark compartment were measured in the training test and in the retention test 24 h later. The maximum entry latency allowed in the retention session was 300 s. A Shut Avoid Vol. 1.8 Harvard apparatus was used for the experiment.

#### 2.3.2. Morris Water Maze Test

The mice at 3, 6, and 12 months of age were subjected to the Morris water maze test to examine their spatial learning ability. The test was conducted as previously described [13]. Mice first received a 3-day habituation period requiring them to swim (60 s) to a visible platform located in a circular tank (diameter, 140 cm; depth, 45 cm) filled with water (22°C) made opaque with powdered milk. Distal visual cues and platform locations were then switched, the platform was submerged (1 cm), and 5 days of trials with the hidden platform ensued. Two hours following hidden platform testing, all mice were given a probe trial (60 s) in which the time spent to reach the target, distance travelled to the target zone, and proximity to the target were recorded. In the hidden platform test, mice were given three trials of 90 s to find the platform (maximum intertrial interval of 45 min), being guided to and allowed to stay on the platform for 5 s on the first day if they exceeded the given time. Measurements were acquired with a Sony camera (model SSC-G18) centrally positioned above the water tank. Behavioral differences were analyzed using the Panlab SMART Video Tracking System v0.3 (Harvard Apparatus). Animals were allowed to dry under heat lamp after each trial to avoid hypothermia, and all experiments were started at the same time each day.

### 2.4. Immunohistochemistry

For immunohistochemical analysis, mice were anesthetized, and transcardiac perfusion was initiated with phosphate-buffered saline (PBS) followed by 4% paraformaldehyde in PBS. Brain tissues from 3-, 6-, and 12-month-old mice were removed and placed in the same fixative overnight at 4°C and then treated sequentially with 10%, 20%, and 30% sucrose in PBS overnight at 4°C. Brains were embedded in OCT and kept at −80°C until further use. The tissue was coronally sectioned (10 *μ*m thick) and collected onto gelatin-coated slides at −20°C using Leica cryostat (CM1850-UV). Tissue sections were stored at −80°C, unless they were immediately used for further immunostaining procedures. The endothelin-1 and oxytocin peptide immunostaining was conducted as described in their product data sheets. Primary antibodies (rabbit anti-endothelin, 1 : 750, Cat. number ab113697, Abcam, and rabbit anti-oxytocin, 1 : 500, Cat. number ab2078, Abcam) and secondary Alexa-Fluor® 488 (Cat. number ab150077, Abcam) were used for immunostaining. Images were obtained with an Olympus BX53 microscope.

### 2.5. Statistical Analysis

All data were processed and analyzed using Excel (Microsoft) software. Quantification of peptide accumulation in hippocampal areas was performed using Fiji software [14]. Statistical analyses with respect to genotype in the same age group of animals in the passive avoidance experiments were tested by two-sample *t-test*s. Quantification and analysis of water maze data were conducted using the video tracking system recording as previously described. For each acquisition trial, the latency (i.e., time in seconds to reach the platform), distance travelled to the platform, and proximity of the animal's position to the goal were measured. Results obtained from the water maze were graphed using a trial version of GraphPad Prism 7. Potential group differences with respect to age and genotype were tested by factorial analysis of variance.

## 3. Results and Discussion

### 3.1. CathA Enzymatic Deficiency Effects on Cognitive Function

**Figure 1:**
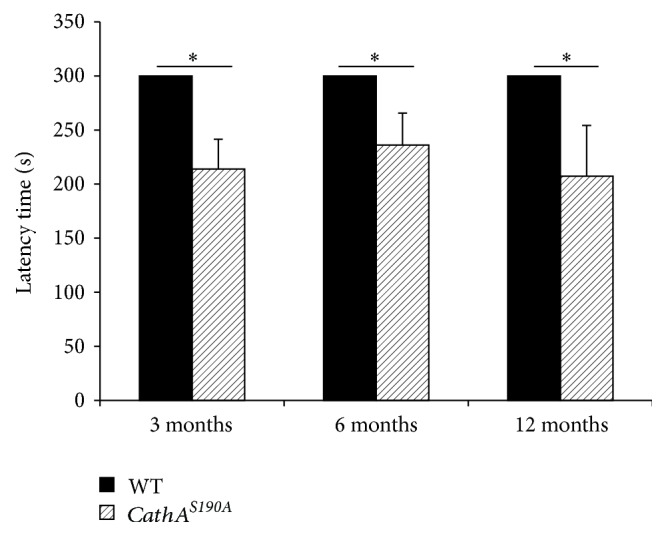
*CathA* ^*S*190*A*^ mice 3, 6, and 12 months old performed significantly worse than their aged-matched wild-type littermates in a passive avoidance task. Latencies to enter the dark compartment on the test day are shown. If mice did not enter the dark compartment within 600 s, a latency time of 600 s was recorded. The data are presented as the mean ± standard error of the mean (SEM); ^*∗*^*p* < 0.05.

The effect of CathA catalytic deficiency and subsequent vasoactive peptide accumulation on memory was investigated using the passive avoidance test (Figure 1). Compared with that of their wild-type littermates, *CathA*^*S*190*A*^ mice took significantly less time to reenter the dark compartment on the test day at 3 months (wild-type: 300 s; *CathA*^*S*190*A*^: 213.96 s ± 27.48; *p* = 0.02; *n* = 6 for both conditions), 6 months (wild-type: 300 s; *CathA*^*S*190*A*^: 236.08 ± 29.46 s; *p* = 0.04; *n* = 8 for both conditions), and 12 months of age (wild-type: 300 s; *CathA*^*S*190*A*^: 207.25 ± 46.93 s; *p* = 0.04, *n* = 8 for both conditions). Mice in the wild-type groups at all ages tested did not enter the dark compartment on test day after having previously received foot shock in that compartment, whereas the *CathA*^*S*190*A*^ mice reentered the chamber, showing the same deterioration in memory function across all age groups.

### 3.2. CathA Enzymatic Deficiency Effects on Spatial Learning and Memory

**Figure 2:**
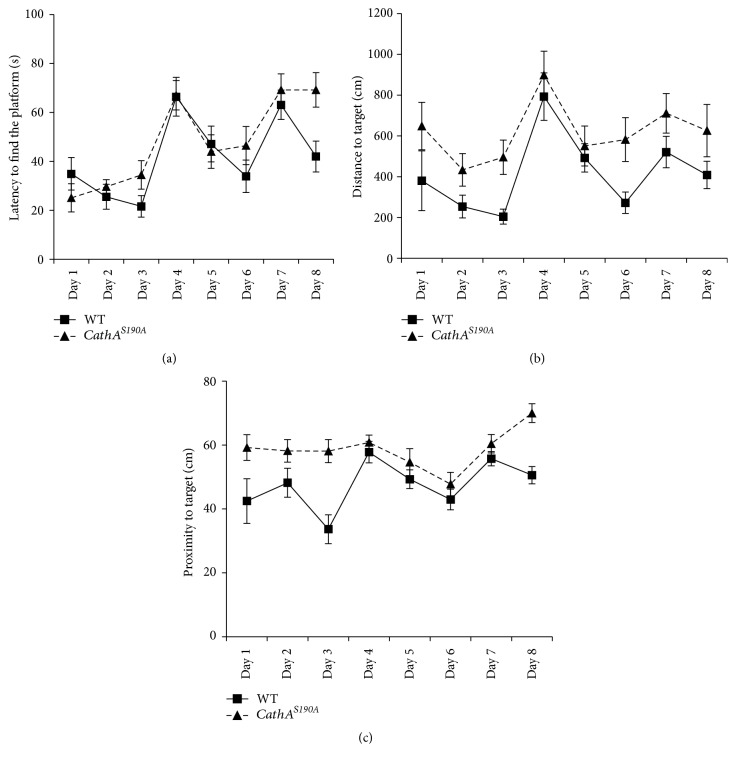
Learning and memory is impaired in 3-month-old *CathA*^*S*190*A*^ mice. Comparison of Morris water maze test results for *CathA*^*S*190*A*^ mice and their littermates at 3 months of age. (a) Average escape latency. (b) Distance moved before reaching the platform. (c) Proximity of the mice to target in the visible platform test (days 1–3) and in the hidden platform test (days 4–8). Graph shows average time or distance ± SEM.

Three-month-old *CathA*^*S*190*A*^ mice displayed spatial learning and memory deficits in the Morris water maze task. Wild-type mice learned to use the visual clues to quickly reach the visible escape platform in the first 3 days, whereas *CathA*^*S*190*A*^ mice took a longer time to swim toward the platform. Both groups initially had difficulty finding the exact location of the platform once it was submerged beneath the opaque water starting on day 4. However, both groups quickly improved in their ability to find the platform. Although wild-type mice oriented themselves better as the daily trials progressed, the ability of *CathA*^*S*190*A*^ mice appeared to decrease over time (*F*_(1.269)_ = 5.97; *p* = 0.01) (Figure 2(a)). In addition to having shorter escape latencies, the wild-type group learned shorter paths to the platform than the *CathA*^*S*190*A*^ mice in the first 3 days of training (Figure 2(b)). Overall, *CathA*^*S*190*A*^ mice swam significantly longer distances than wild-type mice did searching for the platform (*F*_(1.269)_ = 8.06; *p* = 0.005). Proximity to the target measurements also supported that wild-type mice oriented themselves in the pool so that they were significantly closer to the platform in their search (*F*_(1.251)_ = 16.25, *p* < 0.0001; wild-type (*n* = 7) and *CathA*^*S*190*A*^, *n* = 8; Figure 2(c)).

**Figure 3:**
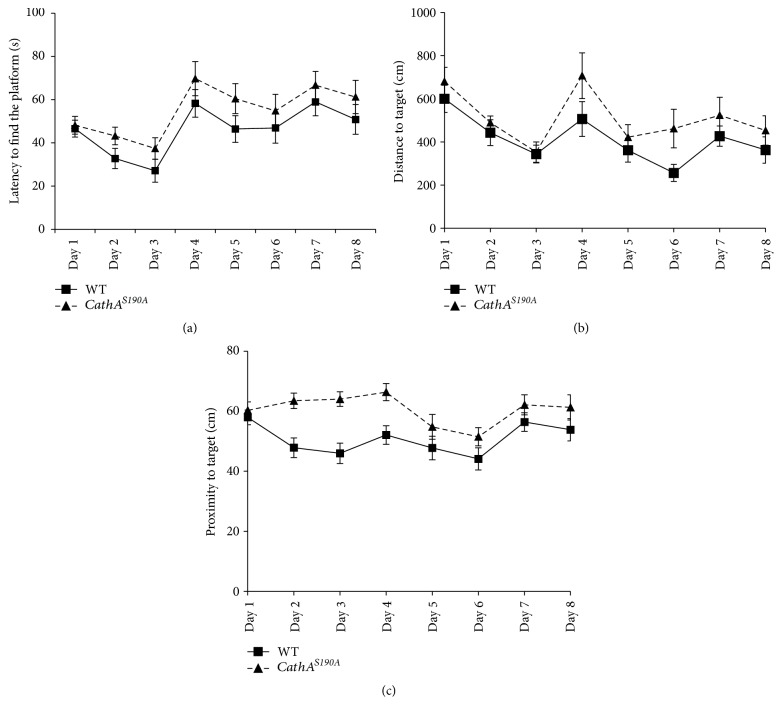
Learning and memory is impaired in 6-month-old *CathA*^*S*190*A*^ mice. Comparison of Morris water maze test results for *CathA*^*S*190*A*^ mice and wild-type littermates at 6 months of age. (a) Average escape latency. (b) Distance travelled before reaching the platform. (c) Proximity of the mice to target in the visible (days 1–3) and in the hidden platform (days 4–8) tests. Graph shows average time or distance ± SEM.

In 6-month-old animals, wild-type and *CathA*^*S*190*A*^ mice showed a parallel learning pattern but a different learning pace. All mice gave similar cognitive reactions to changes of the distal clues. However, *CathA*^*S*190*A*^ mice adapted more slowly than wild-type controls to changing visual patterns (*F*_(1.346)_ = 8.40, *p* = 0.003; Figure 3(a)). The average distance wild-type mice swam to find the platform was significantly less than that for their *CathA*^*S*190*A*^ littermates (*F*_(1.341)_ = 10.47, *p* = 0.001; Figure 3(b)). The proximity to the platform measurements demonstrated that wild-type animals used distal clues more efficiently than *CathA*^*S*190*A*^ mice did to stay nearer to the platform (*F*_(1.382)_ = 20.69, *p* < 0.0001; Figure 3(c)).

**Figure 4:**
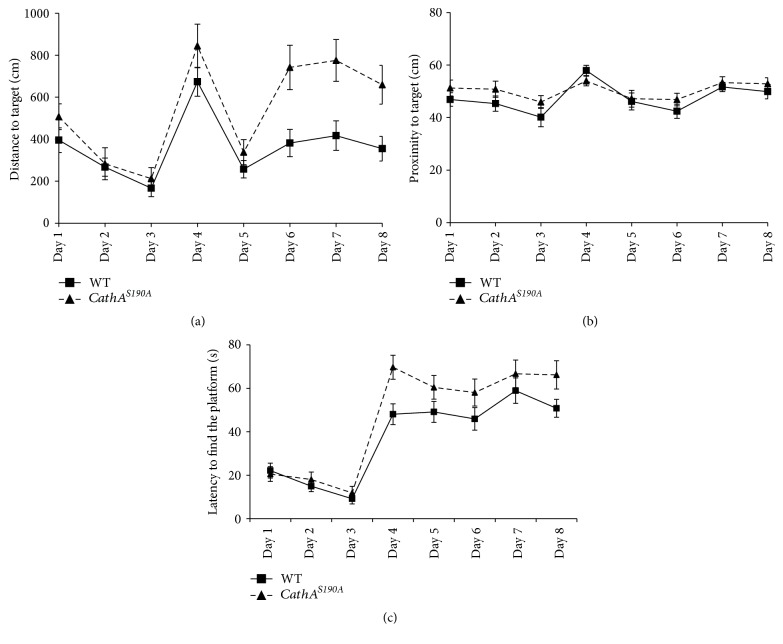
Learning and memory is impaired in 12-month-old *CathA*^*S*190*A*^ mice. Comparison of Morris water maze test results for *CathA*^*S*190*A*^ mice and their wild-type littermates at 12 months of age. (a) Average escape latency. (b) Distance travelled before reaching the platform. (c) Proximity of mice to target in the visible (days 1–3) and in the hidden platform (days 4–8) tests. Graph shows average time or distance ± SEM.

**Figure 5:**
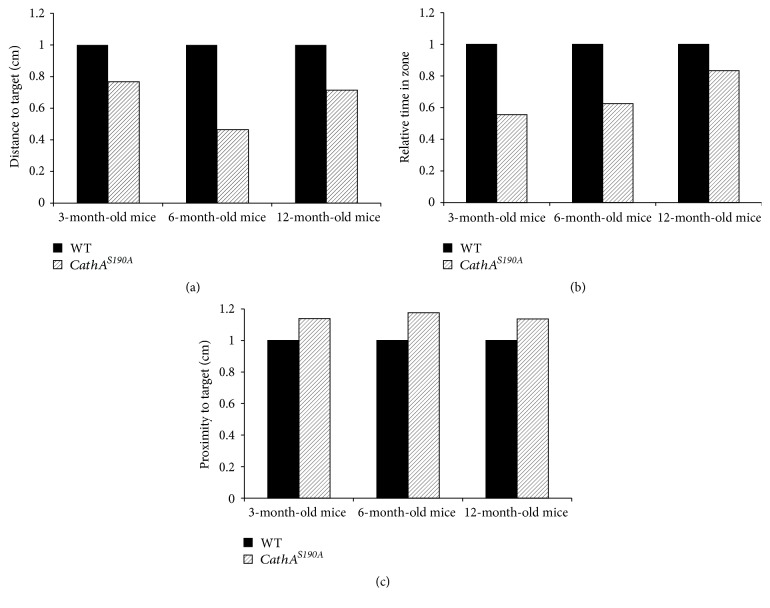
Probe trial in 3-, 6-, and 12-month-old mice. Graph shows relative time spent in the platform area after the platform was removed on day 8. Only the animals that swam on all 8 days were used for the experiment. Wild-type mice were normalized to their *CathA*^*S*190*A*^ counterparts.

At 12 months of age, the escape latency for both groups over the first 3 days of the experiment continuously decreased compared with that of the previous day. The wild-type group adapted better than *CathA*^*S*190*A*^ mice did to the submerged platform on day 4. After this point, although the escape latency scores for the *CathA*^*S*190*A*^ mice improved until day 8, they were consistently lower than those for the wild-type group (*F*_(1.417)_ = 7.90, *p* = 0.005; Figure 4(a)). The 12-month-old wild-type mice swam shorter distance than their *CathA*^*S*190*A*^ counterparts on each day. After day 4, wild-type mice swam direct paths to the platform, whereas their *CathA*^*S*190*A*^ littermates swam significantly longer distances to reach the platform (*F*_(1.413)_ = 21.44, *p* < 0.0001; Figure 4(b)). Despite the proximity results indicating that wild-type and *CathA*^*S*190*A*^ mice swam nearly equal distances from the platform, overall, wild-type mice had significantly better scores in the proximity analysis (*F*_(1.436)_ = 3.98  *p* = 0.04; Figure 4(c)). A probe trial with the platform removed was conducted after animals completed the sessions on day 8 to determine whether the animals found platform location by orienting themselves relative to environmental cues or by coincidence. In this probe trial, both traditional measures of time spent within the platform zone and total distance moved within the platform zone and a relatively new and widely accepted measure of proximity to the goal were analyzed. The analysis of the latency within the zone showed that 3-, 6-, and 12-month-old wild-type mice spent more time than their *CathA*^*S*190*A*^ littermates in the platform zone (Figure 5(a)). The analysis of distance travelled within the zone also showed that *CathA*^*S*190*A*^ mice swam shorter distance in the platform zone than their wild-type counterparts did (Figure 5(b)). In addition to these traditional methods, the measure of proximity during their search for the platform location indicated that wild-type mice spent more of their time closer to the platform position than *CathA*^*S*190*A*^ mice did (Figure 5(c)). These differences were not due to motor function changes, as assessed by rotarod tests (data not shown), in which *CathA*^*S*190*A*^ mice performed as well as their wild-type littermates at 3 and 6 months of age and significantly better than their counterparts at 12 months of age. Based on this as well as the probe trial results, we concluded that *CathA*^*S*190*A*^ mice 3–12 months of age have a learning disability and impaired spatial memory.

### 3.3. Immunohistochemistry

**Figure 6:**
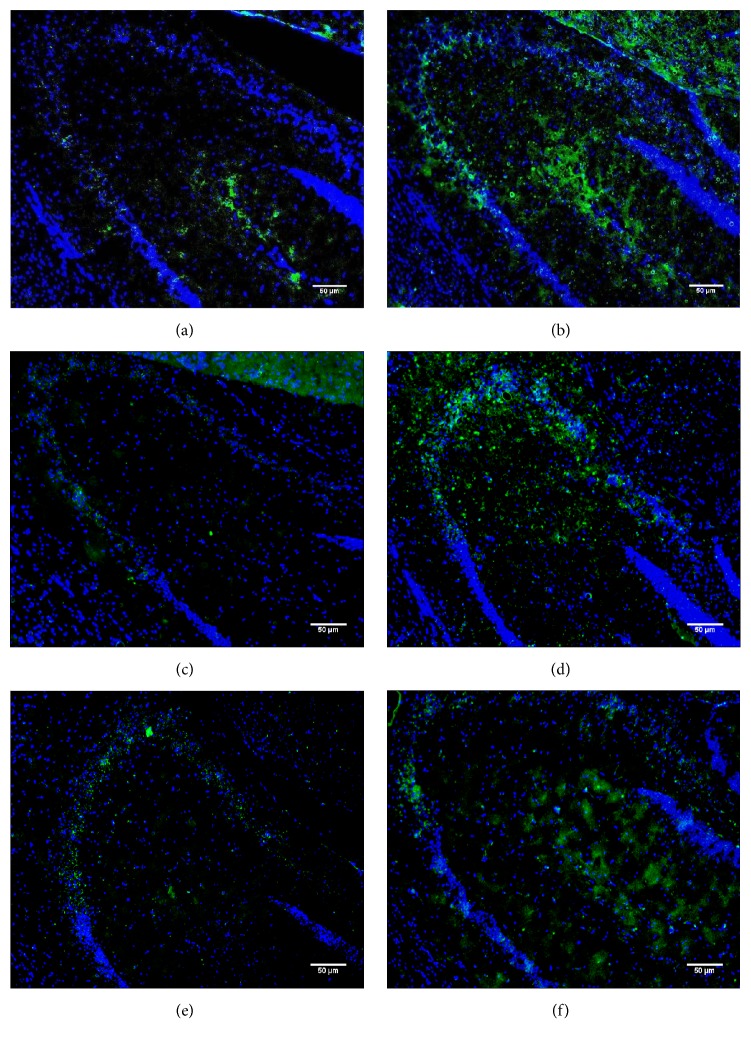
Hippocampal endothelin-1 distribution in 3-, 6-, and 12-month-old wild-type mice and their *CathA*^*S*190*A*^ littermates. Images show the hippocampus of wild-type (a, c, and e) and *CathA*^*S*190*A*^ (b, d, and f) mice after immunostaining with endothelin-1 antibody (green) and DAPI (blue). Images were captured at 20x magnification.

**Figure 7:**
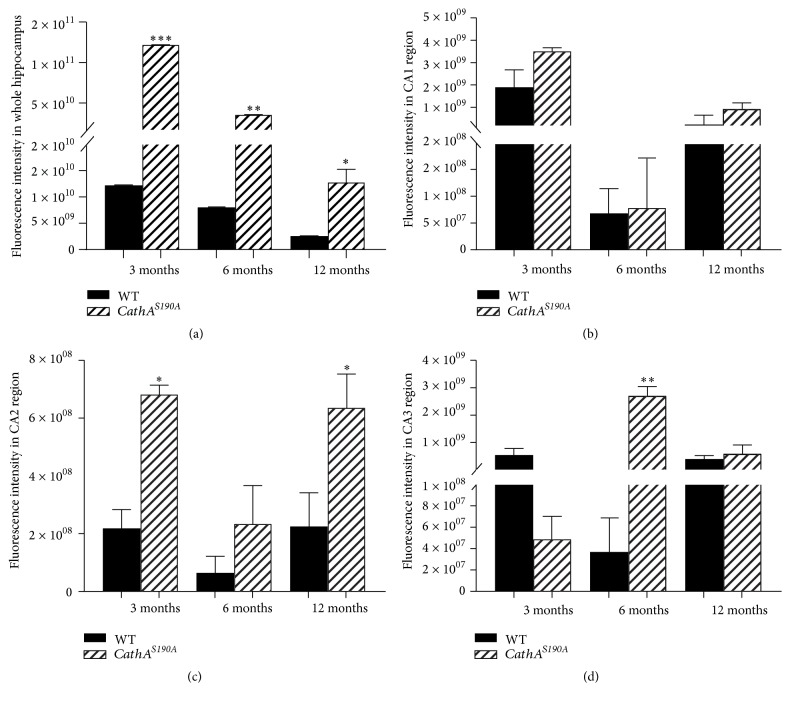
Quantification of hippocampal endothelin-1 accumulation in whole hippocampus (a) and in hippocampal subregions CA1 (b), CA2 (c), and CA3 (d). Six hippocampi from three animals per genotype were analyzed. Graph shows average fluorescence intensity ± SEM.

To understand the reasons underlying the behavioral deficits observed in *CathA*^*S*190*A*^ mice, we characterized the distribution and accumulation of the vasoactive peptides endothelin-1 and oxytocin, which are endogenous substrates for the CathA enzyme. We found that CathA enzyme deficient *CathA*^*S*190*A*^ mice displayed significantly more endothelin-1 accumulation at 3, 6, and 12 months of age than their wild-type mice littermates (Figure 6). Quantification of the fluorescent intensity indicated that accumulation of endothelin-1 was significantly increased at various times in entire hippocampal region of the *CathA*^*S*190*A*^ mouse brain compared with that of their wild-type littermates at 3 (^*∗∗∗*^*p* = 0.004), 6 (^*∗∗*^*p* = 0.01), and 12 (^*∗*^*p* = 0.05) months of age (Figure 7(a)). Significant increases were observed in the CA2 region of *CathA*^*S*190*A*^ mice at 3 months (^*∗*^*p* = 0.04; Figure 7(b)) and 12 months (^*∗*^*p* = 0.05; Figure 7(c)) of age and in the CA3 region at 6 months (^*∗∗*^*p* = 0.008; Figure 7(d)).

**Figure 8:**
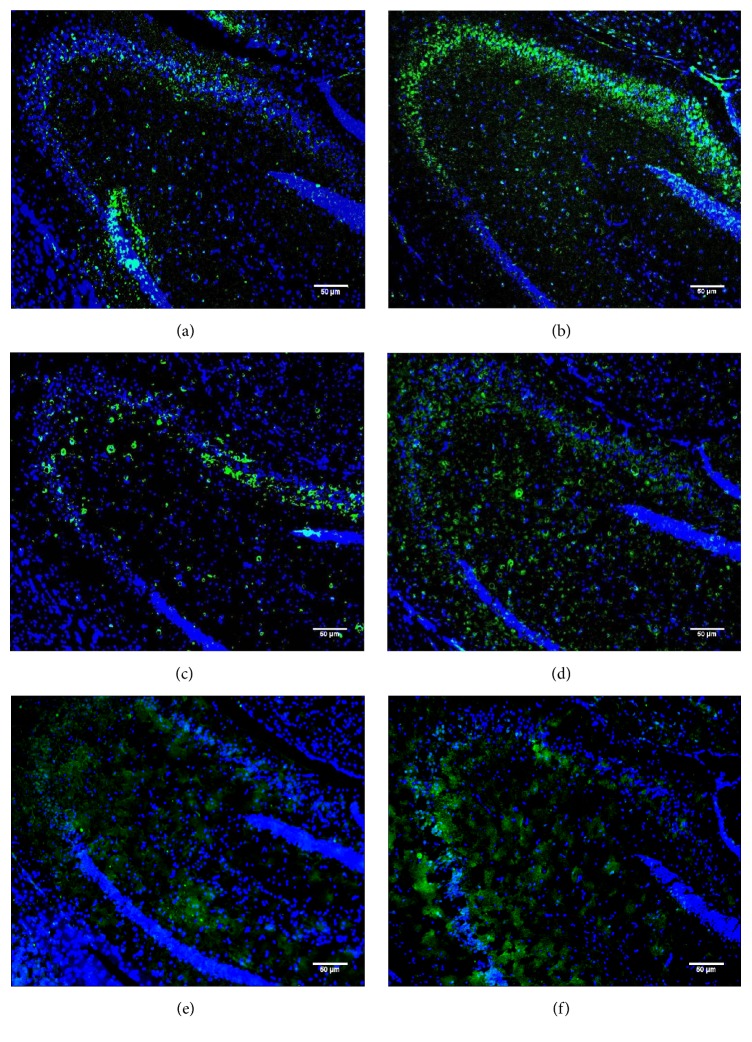
Hippocampal oxytocin distribution in 3-, 6-, and 12-month-old wild-type (a, c, and e, resp.) and *CathA*^*S*190*A*^ (b, d, and f, resp.) mice. Images show the hippocampus of wild-type and *CathA*^*S*190*A*^ mice after immunostaining with an oxytocin antibody (green) and DAPI (blue). Images were captured at 20x magnification.

**Figure 9:**
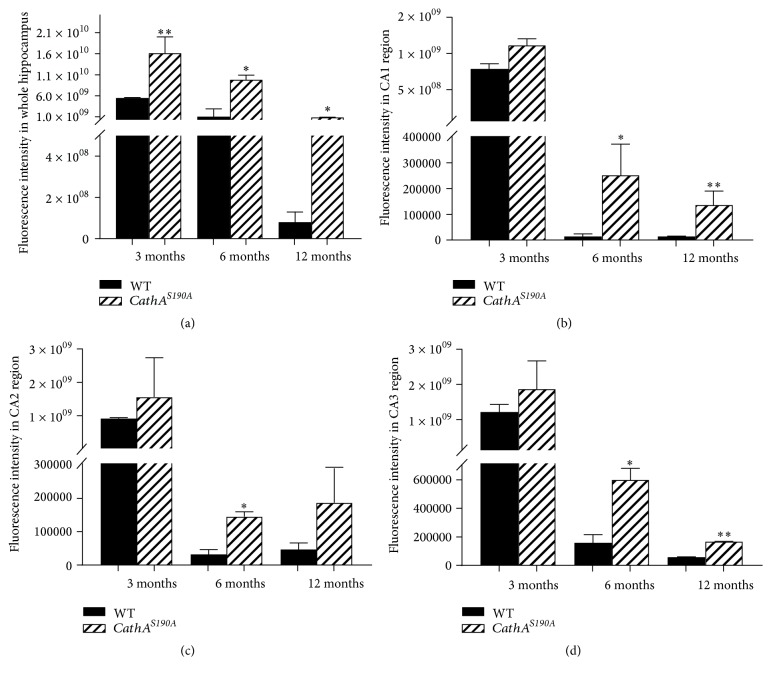
Quantification of hippocampal oxytocin accumulation in whole hippocampus (a) and hippocampal subregions CA1 (b), CA2 (c), and CA3 (d). Six hippocampi from three mice per genotype were analyzed. Graph shows average fluorescence intensity ± SEM.

Analysis of the oxytocin distribution indicated a clear accumulation of oxytocin in 3-, 6-, and 12-month-old *CathA*^*S*190*A*^ mice (Figures 8(b), 8(d), and 8(f)) compared with that in their wild-type littermates (Figures 8(a), 8(c), and 8(e)). The quantification results showed significantly increased oxytocin immunostaining in the whole hippocampus of *CathA*^*S*190*A*^ mice at 3 (^*∗∗*^*p* = 0.01), 6 (^*∗*^*p* = 0.04), and 12 (^*∗*^*p* = 0.05) months of age (Figure 9(a)). At 6 months old, *CathA*^*S*190*A*^ mice showed oxytocin accumulation in hippocampal CA1 (^*∗*^*p* = 0.02), CA2 (^*∗*^*p* = 0.03), and CA3 (^*∗*^*p* = 0.05) regions (Figures 9(b), 9(c), and 9(d)). In 12-month-old animals, significant accumulation was detected in CA1 (^*∗∗*^*p* = 0.01) and CA3 (^*∗∗*^*p* = 0.06) (Figures 9(b) and 9(d)).

## 4. Conclusion

In this study, changes in learning and spatial memory were investigated in *CathA*^*S*190*A*^ mice to assess the impact of the absence of the CathA catalytic function on the processing of two vasoactive peptides: endothelin-1 and oxytocin.

*CathA* ^*S*190*A*^ mice 3, 6, and 12 months of age were more willing than their wild-type littermates to enter the dark chamber in the shuttle box test, despite previous foot shocks delivered there during passive avoidance tests. This could be due to deficits in their inability to create fear-associated memories. In addition, a spatial memory deficit was observed in the Morris water maze task in the *CathA*^*S*190*A*^ mice at all three ages. The hippocampus is an important brain region governing cognitive and memory functions. Lesions, long-term potentiation problems, such as increased levels of vasoactive peptides, and loss-of-function genetic mutations of signaling molecules/receptors within the hippocampus lead to impairments in spatial learning and memory. The increase in escape latency, distant travelled to the platform, and proximity to target measurements observed in the *CathA*^*S*190*A*^ mice as well as their failure to spend more time in the correct quadrant during the probe trial compared with their wild-type littermates suggested that a deficiency in CathA enzyme activity leads to an impairment in spatial learning and memory.

Previous in vitro analyses revealed the hydrolytic activity of CathA against various short peptides, including endothelin-1 and oxytocin [14]. Endothelin-1 and oxytocin have a broad range of roles in the regulation of cellular pathways promoting long-term potentiation and long-lasting spatial memory with CREB phosphorylation in the hippocampus [15, 16]. Therefore, we investigated whether elevation of these peptides, as a result of abolishing CathA hydrolytic enzyme activity, was related to the learning and memory deficits studied in the *CathA*^*S*190*A*^ mouse model.

Our immunohistochemistry results indicated that endothelin-1 accumulated in hippocampus of *CathA*^*S*190*A*^ mice, consistent with the previously reported distribution of this peptide in the brain tissue of galactosialidosis patients [17] and western blot analysis results derived from knockout animal models [9]. In the central nervous system CathA has strong peptidase activity [18]. We confirmed that endothelin-1 is a substrate for CathA and then showed, for the first time, region-specific accumulation of the peptide in hippocampus of 3-, 6-, and 12-month-old *CathA*^*S*190*A*^ mice. To our knowledge, we are also the first to show that at physiological levels oxytocin is another substrate for CathA, the deficiency of which causes accumulation of oxytocin in the hippocampus.

The present study suggests an intriguing possibility of using endothelin-1 and oxytocin, as well as their therapeutic analogs, as novel therapeutic targets for memory loss, such as that seen in Alzheimer's disease. If the role of these peptides in memory improvement is confirmed, development of new medical approaches to introduce oxytocin and endothelin-1 into the central nervous system may be beneficial in the treatment of memory loss-related diseases. In addition, as discovery of new substrates of CathA could be used as possible biomarkers in the treatment of cardiovascular diseases, such as hypertension, results from these studies will allow better understanding of lysosomal enzyme-based therapeutic approaches.

## References

[1] Galjart N. J., Morreau H., Willemsen R., Gillemans N., Bonten E. J., d'Azzo A. (1991). Human lysosomal protective protein has cathepsin A-like activity distinct from its protective function. *Journal of Biological Chemistry*.

[2] Pshezhetsky A. V. (2013). Lysosomal carboxypeptidase A. *Handbook of Proteolytic Enzymes*.

[3] Skidgel R. A., Erdös E. G. (1998). Cellular carboxypeptidases. *Immunological Reviews*.

[4] Bonten E. J., Annunziata I., D'Azzo A. (2014). Lysosomal multienzyme complex: pros and cons of working together. *Cellular and Molecular Life Sciences*.

[5] Hiraiwa M. (1999). Cathepsin A/protective protein: an unusual lysosomal multifunctional protein. *Cellular and Molecular Life Sciences*.

[6] D'Azzo A., Hoogeveen A., Reuser A. J., Robinson D., Galjaard H. (1982). Molecular defect in combined beta-galactosidase and neuraminidase deficiency in man. *Proceedings of the National Academy of Sciences of the United States of America*.

[7] Cuervo A. M., Mann L., Bonten E. J., d’Azzo A., Dice J. F. (2003). Cathepsin A regulates chaperone‐mediated autophagy through cleavage of the lysosomal receptor. *The EMBO Journal*.

[8] Ashmarin I. P., Buzinova E., Vinogradova M., Potier M., Pshezhetsky A. (1997). Learning disability in rats induced by the immunosuppression of cathepsin A in blood plasma. *Neuroscience Research Communications*.

[9] Zhou X. Y., Morreau H., Rottier R. (1995). Mouse model for the lysosomal disorder galactosialidosis and correction of the phenotype with overexpressing erythroid precursor cells. *Genes & Development*.

[10] Seyrantepe V., Hinek A., Peng J. (2008). Enzymatic activity of lysosomal carboxypeptidase (cathepsin) a is required for proper elastic fiber formation and inactivation of endothelin-1. *Circulation*.

[11] Deacon R. M. J. (2013). Measuring motor coordination in mice. *Journal of Visualized Experiments*.

[12] Schindelin J., Arganda-Carreras I., Frise E. (2012). Fiji: an open-source platform for biological-image analysis. *Nature Methods*.

[13] Seyrantepe V., Lema P., Caqueret A. (2010). Mice doubly-deficient in lysosomal hexosaminidase a and neuraminidase 4 show epileptic crises and rapid neuronal loss. *PLoS Genetics*.

[14] Jackman H. L., Tan F., Tamei H. (1990). A peptidase in human platelets that deamidates tachykinins: probable identity with the lysosomal ‘protective protein’. *Journal of Biological Chemistry*.

[15] Kuwaki T., Kurihara H., Cao W. H. (1997). Physiological role of brain endothelin in the central autonomic control: from neuron to knockout mouse. *Progress in Neurobiology*.

[16] Tomizawa K., Iga N., Lu Y.-F. (2003). Oxytocin improves long-lasting spatial memory during motherhood through MAP kinase cascade. *Nature Neuroscience*.

[17] Itoh K., Oyanagi K., Takahashi H. (2000). Endothelin-1 in the brain of patients with galactosialidosis: its abnormal increase and distribution pattern. *Annals of Neurology*.

[18] Sohma O., Mizuguchi M., Takashima S. (1999). Expression of protective protein in human tissue. *Pediatric Neurology*.

